# Progress in understanding the role of lncRNA in programmed cell death

**DOI:** 10.1038/s41420-021-00407-1

**Published:** 2021-02-08

**Authors:** Na Jiang, Xiaoyu Zhang, Xuejun Gu, Xiaozhuang Li, Lei Shang

**Affiliations:** Jiangxi Research Institute of Ophthalmology and Visual Sciences, Affiliated Eye Hospital of Nanchang University, Nanchang, 330006 China

**Keywords:** Cell biology, Molecular biology

## Abstract

Long non-coding RNAs (lncRNAs) are transcripts longer than 200 nucleotides but not translated into proteins. LncRNAs regulate gene expressions at multiple levels, such as chromatin, transcription, and post-transcription. Further, lncRNAs participate in various biological processes such as cell differentiation, cell cycle regulation, and maintenance of stem cell pluripotency. We have previously reported that lncRNAs are closely related to programmed cell death (PCD), which includes apoptosis, autophagy, necroptosis, and ferroptosis. Overexpression of lncRNA can suppress the extrinsic apoptosis pathway by downregulating of membrane receptors and protect tumor cells by inhibiting the expression of necroptosis-related proteins. Some lncRNAs can also act as competitive endogenous RNA to prevent oxidation, thereby inhibiting ferroptosis, while some are known to activate autophagy. The relationship between lncRNA and PCD has promising implications in clinical research, and reports have highlighted this relationship in various cancers such as non-small cell lung cancer and gastric cancer. This review systematically summarizes the advances in the understanding of the molecular mechanisms through which lncRNAs impact PCD.

## Facts

- Programmed cell death refers to controlled cell death to maintain the stability of the internal environment.
- LncRNAs are a class of RNA molecules that are longer than 200 nucleotides that control gene expression at the epigenetic, transcription, RNA splicing, and post-transcriptional levels.
- LncRNA can regulate programmed cell death.
- Targeting lncRNA may reduce or reinforce cellular damage caused by lncRNA.

## Open questions

- What is the mechanism of action of lncRNA on different types of programmed cell death?
- How is lncRNA involved in the progression of programmed cell death?
- How can we target lncRNAs to modulate programmed cell death mechanisms?

## Background

Programmed cell death (PCD) is mediated by several cellular gene expression events, which are mainly divided into apoptosis, autophagy, necroptosis, and ferroptosis^1^. Caspase is a key factor mediating apoptosis, which can mediate exogenous and intrinsic pathways and thus lead to apoptosis^2,3^. When caspase activity is inhibited, death receptors (such as Tumor Necrosis Factor [TNF] Receptors [TNFRs]) are activated, followed by activation of receptor-interacting protein kinase 1 (RIPK1), RIPK3, and the mixed lineage kinase domain-like (MLKL) to form necrotic bodies that finally mediate necroptosis^1,4,5^. The main cause of ferroptosis is the accumulation of iron-dependent reactive oxygen species (ROS) and the consumption of plasma membrane polyunsaturated fatty acids^6,7^. Among them, major autophagy is the most important type of autophagy^8^. Under conditions such as starvation, hypoxia, and hormone signaling, cells form autophagosomes that combine with lysosomes, thereby degrading macromolecular substances and causing macroautophagy^9,10^.

PCD plays an important role in human disease and aging^11,12^. There are many factors that affect PCD processes, including altered protein levels and other macromolecular substance dysfunction, abnormal glucose levels, mitochondrial dysfunction, and oxidative stress^13,14^. Recent studies have found that many small molecules, such as lncRNAs, can also regulate PCD in addition to the aforementioned protein factors.

LncRNA is a special RNA type defined as transcripts over 200 nucleotides long, but is not translated into proteins^15^. LncRNAs are numerous and abundant, and were originally considered to be biologically non-functional transcriptional noise. Although lncRNA is not translated into protein, it is still considered a functional molecule that regulates gene expression at multiple levels, including the chromatin, transcriptional, and post-transcriptional levels. Indeed, lncRNA is involved in cell differentiation, cell cycle regulation, stem cell pluripotency, and maintenance of various biological processes^16–19^. LncRNAs can affect different apoptotic pathways. For instance, lncRNA overexpression can reduce the expression of membrane surface receptors by affecting extrinsic apoptosis pathway^20,21^. Autophagy not only promotes cell survival but also causes cell death in some conditions. LncRNA can activate autophagy by activating related enzymes^22^. In addition, lncRNAs can protect tumor cells from necroptosis by inhibiting the expression of some related proteins^20^. Some lncRNA types also act as competitive endogenous RNAs to prevent oxidation and inhibit ferroptosis^20^. Increasing evidence shows that lncRNAs are closely related to PCD, and the relationship between lncRNA and PCD is associated with the occurrence of heart disease, cancer cell apoptosis, and cell survival^23,24^. In this article, we summarize the role of lncRNAs in PCD to determine the relationship between lncRNAs and PCD.

### The relationship between lncRNA and apoptosis

**Fig. 1:**
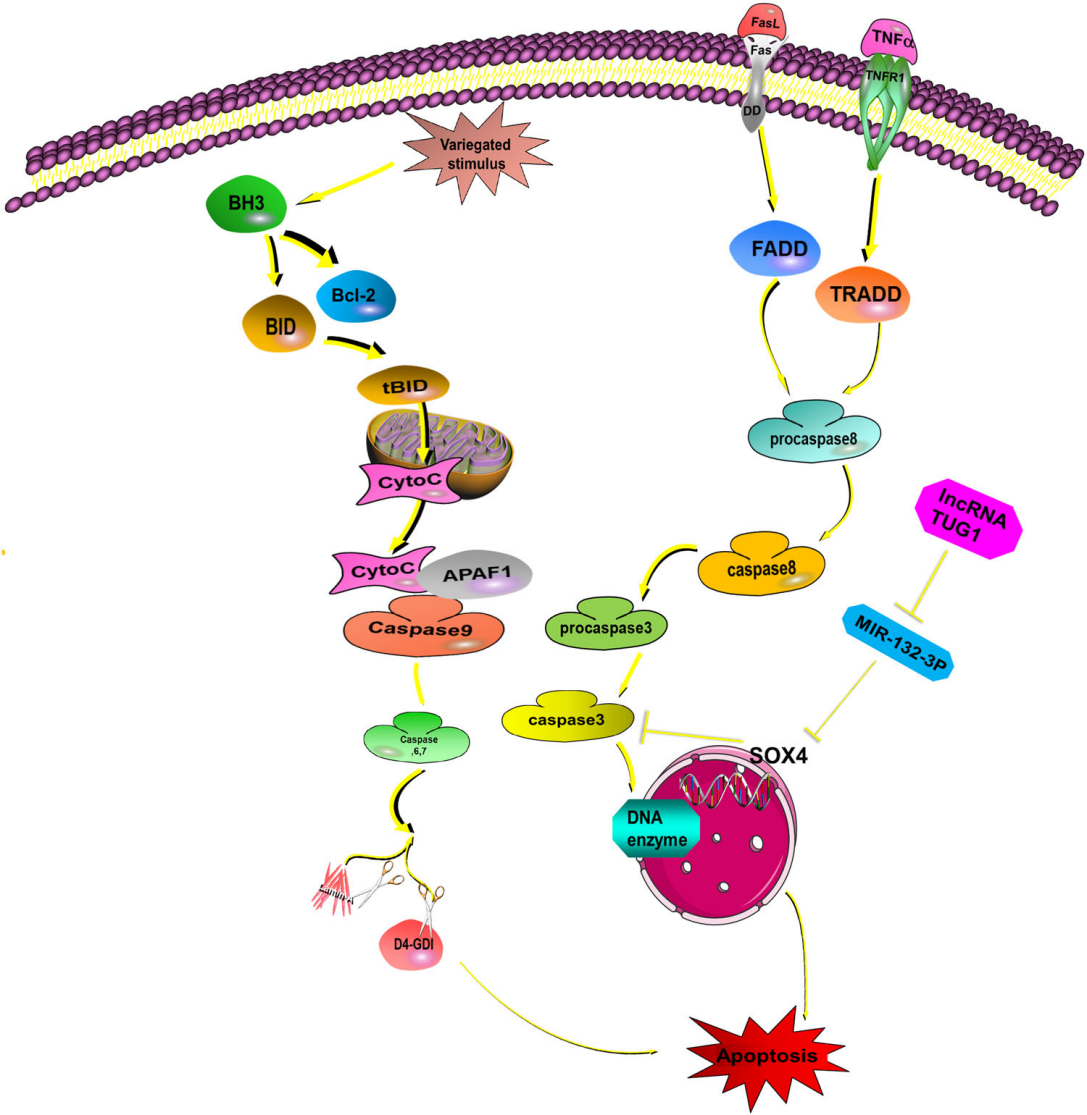
Activation of the apoptotic pathway. Apoptosis can be divided into the extrinsic pathway and intrinsic pathway. In the extrinsic pathway, the death receptor TNFR, Fas/CD95, and TRAIL combine with the relevant factors from extracellular, such as TNF, FasL. Then, the death receptor, procaspase-8, caspase-8, and caspase-3 are sequentially activated and regulate DNA fragmentation factor (DFF)/caspase-activated DNase (CAD) and cause apoptosis^26,27,36^. In the intrinsic pathway, cytotoxic stimuli such as carcinogenic stress, chemotherapeutic agents, and developmental cues activate BH3-only family members and inhibit Bcl-2 proteins, thus activating the pro-apoptotic effectors BAX and BAK, which finally destroy the outer mitochondrial membrane. This releases cytochrome C and ATF endonuclease. Cytochrome C engages APAF1 and forms the apoptosome, which activates caspase-9. Subsequently, caspase-9 activates caspase-3, caspase-6, and caspase-7, which contribute to the cleavage of other proteins and eventually promote apoptosis. Various lncRNAs are involved in this process. For instance, TUG1 can inhibit miR-132-3p expression, thereby increasing SOX4 expression. This decreases caspase-3 activity, eventually inhibiting osteosarcoma (OS) cell apoptosis. This indicates that knockdown of TUG1 could regulate the miR-132-3p–SOX4 axis in OS cells and promote apoptosis.

Apoptosis is initiated by many ligands, which regulate specific membrane death receptors (the extrinsic pathway) and/or the Bcl-2 protein family in mitochondria (the intrinsic pathway)^25^. The extrinsic pathway passes signals from outside the cell to the inside and is regulated by the death receptor TNFR, Fas/CD95, and TNF-related apoptosis-inducing ligand (TRAIL)^26^. Membrane death receptor activation causes procaspase-8 hydrolysis and activation to form activated caspase-8, which then activates downstream caspase-3. Subsequently, DNA fragmentation factor (DFF) subunit/CAD (DNase) is activated, leading to apoptosis^27,28^. In the intrinsic pathway, cytotoxic stimuli such as carcinogenic stress, chemotherapeutic agents, and developmental cues activate BH3-only family members and inhibit Bcl-2 proteins before survival, thus activating the pro-apoptotic effectors BAX and BAK, which finally destroy the outer mitochondrial membrane^29^. After the outer membrane of mitochondria is damaged, cytochrome C and ATF endonuclease are released^30,31^. Cytochrome C engages the apoptotic protease activating factor-1 (APAF1) and forms apoptosomes, which activate caspase-9^32^. Afterward, caspase-9 activates caspase-3, caspase-6, and caspase-7, which contribute to the cleavage of other proteins and finally result in apoptosis^1^ (Fig. 1).

LncRNA can influence apoptosis in several ways. First, lncRNAs can directly or indirectly act on death receptors. We previously reported trophoblast invasion and trophoblast‐mediated VSMC loss in uterine spiral artery remodeling. In that study, we found that extravillous trophoblasts (EVTs) can generate TNF-α, Fas ligand (FasL), and TRAIL. These are important factors mediating EVT-induced VSMC loss, and the combination with VSMC membrane receptors results in VSMC apoptosis. We used lncRNA MEG3-overexpressing plasmid to transfect trophoblast HTR‐8/SVneo cells to increase MEG3 expression. qRT-PCR and Western blotting showed that MEG3 overexpression reduced TNF-α, FasL, and TRAIL expression. Next, VSMCs and transfected HTR-8/SVneo cells were co-cultured for 48 h, and the apoptosis rate of VSMCs was detected by flow cytometry. The results showed that compared with the negative control group, the apoptosis rate of VSMCs in the H‐MEG3 group was significantly reduced, indicating that MEG3 upregulation in HTR‐8/SVneo cells inhibited VSMC apoptosis. These results indicate that MEG3 might be a negative regulator of spiral artery remodeling by acting on the apoptosis-related death receptor, thereby suppressing EVT-mediated VSMC apoptosis^20^.

Second, lncRNA can act as competitive endogenous RNA (ceRNA) for miRNA by binding with a sequence at the miRNA 5′ end, thereby decreasing target mRNA expression and finally influencing cell apoptosis. A study of osteogenic sarcoma (OS) showed that the lncRNA, Taurine upregulates gene1 (TUG1), regulates disease development. Indeed, TUG1 is overexpressed in OS, potentially targeting miR-132-3p. Luciferase reporter gene analysis and qRT-PCR analysis showed that TUG1 directly binds at its 3′-terminus “-GACUGUUG-” sequence with the miR-132-3p 5′-terminus “-CUGACAA-” sequence, thereby inhibiting miR-132-3p expression, decreasing caspase-3 activity, and eventually inhibiting OS cell apoptosis. Moreover, miR-132-3p inhibitors significantly decreased the apoptosis rate and caspase-3 activity, indicating that miR-132-3p induces apoptosis. The sex-determined region Y-frame 4 (SOX4) is a known oncogene^33,34^ and a target of miR-132-3p in OS cells. Introducing SOX4 partially reverses the apoptosis-promoting effect of miR-132-3p. This indicates that TUG1 knockdown could regulate the miR-132-3p–SOX4 axis in OS cells and promote apoptosis^21^.

These studies indicate that lncRNAs can regulate apoptosis *via* various mechanisms^35,36^. For instance, Zhang et al. demonstrated that lncRNA TUG1 inhibits apoptosis by downregulating miR-29b^37^. After LPS treatment, the expression of apoptosis-inducing caspase 3 and cytochrome C in H9c2 cells increased. In addition, qRT-PCR and ELISA showed that the expression of inflammatory factors such as IL-6, IL-8, and TNF-α increased after LPS treatment. These results show that exposure to LPS induces H9c2 cells apoptosis and induces inflammation. Flow cytometry analysis showed that the apoptosis rate in PEx-Tug1-transfected H9c2 cells was significantly reduced following LPS treatment, while TUG1 inhibition significantly increased H9c2 cell apoptosis. With the decrease in apoptosis rate, TUG1 overexpression downregulated caspase 3 and cytochrome C expression, and inhibited IL-6, IL-8, and TNF-α expression and secretion, while TUG1 inhibition enhanced the levels of these cytokines. Overall, these results demonstrate that TUG1 has a protective effect against LPS-induced damage in H9c2 cells. In addition, TUG1 overexpression significantly downregulates miR-29b, while TUG1 inhibition upregulates miR-29b. Based on these results, there may be binding sites between TUG1 and miR-29b: the 5′end “-AACACTGA-” of TUG1-wt could bind to the 3′end “-UUGUGACU-” of miR-29b.

Furthermore, TUG1 also inhibits NF-κB signaling and the JAK/STAT pathway by downregulating miR-29b to induce inflammatory responses and apoptosis. CCK-8 assays showed that LPS exposure in H9c2 cells increases p65 and IκBα phosphorylation, while TUG1 overexpression inhibits NF-κB pathway activation. However, miR-29b mimics reverse TUG1 overexpression by reducing p-p65 and p-IκBα expression. Similar changes were observed in the JAK/STAT pathway. LPS stimulation increase s p-JAK and p-STAT3 expression, while TUG1 overexpression reduces their expression. In addition, miR-29b mimics block TUG1-mediated inhibition of the JAK/STAT pathway. These findings suggest that TUG1 overexpression inactivates the NF-κB and JAK/STAT pathways by downregulating miR-29b.

Similarly, a study of the lncRNA Highly Upregulated in Liver Cancer (HULC) showed that restoring HULC expression could rescue TNF-induced apoptosis, and that HULC regulates TNF-induced apoptosis by regulating miR-9 expression^38^. Moreover, A study of osteoarthritis demonstrated that knocking down the lncRNA reprogramming (ROR) can inhibit chondrocyte apoptosis through HIF1 (Hypoxia-inducible factors 1) and p53^39^.

In summary, lncRNAs can affect apoptosis in various ways, such as by acting on apoptosis-related receptors or by acting as ceRNAs.

### The relationship between lncRNA and autophagy

Autophagy promotes cell survival, but can also cause cell death. Autophagy usually occurs in three forms: macroautophagy, microautophagy, and CMA^8^.

### Macroautophagy

Macroautophagy is a conserved process in eukaryotic cells, and is the most studied form of autophagy. In this process, the phagocyte membrane envelops the cytoplasmic contents, building a double membrane structure called an autophagosome^40^. The autophagosome then fuses with a lysosome, which exposes the contents to lysosomal hydrolases. Next, the autophagosome endomembrane and enclosed macromolecular substances degrade and the substances are released into the cytoplasm *via* the lysosomal membrane osmotic enzyme and are used to synthesize new macromolecules.

**Fig. 2:**
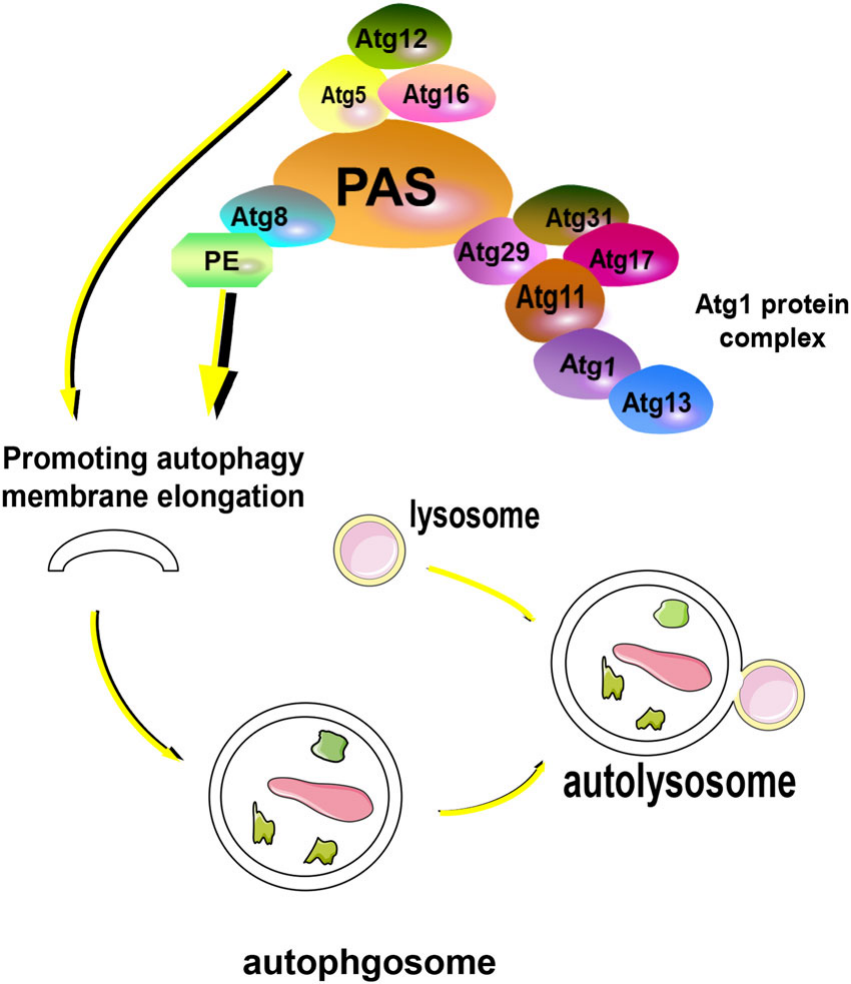
Autophagosome integration in yeast cells. First, binding with Atg31 causes Atg29 to integrate into the Atg17–Atg31–Atg29 complex. Atg29 phosphorylation plays a key role in binding with other phosphoproteins. Moreover, Atg29 and Atg11 directly interact. Eventually, Atg1 combines with Atg13 and Atg11, with the latter directly associating with Atg17 and Atg19. In this way, the Atg1 protein complex takes shape. Moreover, the atg12–atg5–atg16 complex moves to the PAS and makes Atg8 combine with phosphatidylethanolamine (PE) to form the Atg8–PE complex, which promotes the elongation of the autophagosome membrane and forms autophagosomes and autolysosomes.

In yeast cells, for example, autophagosomes are constructed at the phage assembly site (PAS) and are necessary for the whole process. The ATG (Autophagy Related) gene is involved in autophagosome formation, and when macroautophagy is induced, Atg proteins are recruited to promote autophagosome biogenesis^41^. The Atg1 protein complex plays an important role in recruiting other Atg proteins to the PAS. Several subunits of the complex are phosphoproteins, including Atg1 protein kinase, Atg13, Atg11, and Atg17–Atg31–Atg29 (sub) complexes. The integration process is as follows: First, by binding with Atg31, Atg29 correctly integrates into the Atg17–Atg31–Atg29 complex. Atg29 phosphorylation plays a key role in binding with other phosphoproteins^42,43^. Moreover, Atg29 directly interacts with Atg11. Eventually, Atg1 combines with Atg13 and Atg11, and then associates directly with Atg17 and Atg19. Thus, the Atg1 protein complex forms^44^. Then, the Atg1 protein complex combines and phosphorylates Atg9 on vesicles and then recruits PI3KC3–C1 (ATG34:ATG15:ATG6:ATG14) to the PAS (Fig. 2). Additionally, when the Atg12–atg5–atg16 complex moves to the PAS, Atg8 combines with phosphatidylethanolamine (PE) to form the Atg8–PE complex, which promotes autophagosome membrane elongation, autophagosome formation, and autolysosome formation^45^. Kotani et al. explain the initiation of membrane expansion in the formation of autophagosomes with the following model: amphiphilic spirals in the Atg2 and Atg18 C-terminal regions cooperate to bind Atg9 vesicles on the PAS, and the Atg2 N-terminal region binds to the endoplasmic reticulum (ER)^46^. These interactions lead to PAS–ER binding, resulting in membrane expansion and autophagosome formation.

### Microautophagy

Microautophagy is another type of autophagy that occurs *via* vacuolar phagocytosis, incorporation of organelle components (including organelles), and transport into the lysosome or vacuole system^47,48^. In yeast cells, microautophagy degrades the proteins and organelles in cells contained within lysosomes or vacuoles. That this degradation has a discriminant capacity, and even portions of the nucleus can be degraded in this way^47,49^.

### Chaperone-mediated autophagy (CMA)

In contrast to other processes, in CMA, the substrate enters the lysosome lumen *via* the protein transport complex on the lysosome membrane. Additionally, this process directs targeted cytoplasmic proteins to the lysosome^50^. Moreover, CMA is selective, and the organelles, lipids, nucleic acids, or integral membrane proteins are not degraded^51^. CMA responds to stress responses such as hunger, lipid toxicity, protein toxicity, hypoxia, oxidative stress, or DNA damage^52^. By degrading specifically targeted cell proteins, CMA can regulate glucose and lipid metabolism, DNA repair, cellular reprogramming, and cellular responses to stress^53^.

There are few studies on microautophagy and CMA. Therefore, we will mainly discuss how lncRNA is involved in macroautophagy (hereinafter referred to as autophagy).

## lncRNA MALAT1

A 2019 study showed that MALAT1 overexpression recruits Enhancer of Zeste 2 Polycomb repressive complex 2 subunit (EZH2) to the Tuberous sclerosis 2 (TSC2) promoter region, which upregulates H3K27me3 histones, resulting in the inhibition of autophagy^54^. Contrastingly, lncRNAs also promote the occurrence of autophagy in tumor cells^55^.

Another study showed that MALAT1 overexpression activates autophagy in colorectal cancer (CRC). The results showed that MALAT1 expression in CRC was very high compared to expression in adjacent normal tissues. MALAT1 expression was greatly reduced by si-MALAT1 transfection, while MALAT1 expression was increased by pcDNA-MALAT1. Western blotting showed that MALAT1 downregulation reduced LC3-II/LC3-I (light chain) expression and increased p62 (a protein provided by SQSTM1 gene) expression. However, MALAT1 upregulation has the opposite effect. This indicates that MALAT1 is related to autophagy activation. In the cells inactivated by MALAT1, caspase-3 expression was increased significantly. Similarly, MALAT1 upregulation resulted in decreased caspase-3 cleavage compared with the pcDNA transduction group. There was no difference in caspase-9 expression between the two groups. Flow cytometry showed that the apoptosis rate of the si-MALAT1 group was significantly higher than in the siRNA group. At the same time, MALAT1 upregulation reduced the apoptosis rate. In addition, MALAT1 upregulation was reduced by the autophagy inhibitor 3-mA. These data show that MALAT1 can increase cell proliferation and inhibit CRC cell apoptosis by activating autophagy.

Importantly, miR-101 and MALAT1 have complementary sequences, suggesting that miR-101 is a target of MALAT1. Compared with adjacent normal tissues, miR-101 expression in CRC was very high, and MALAT1 and miR-101 expression were negatively correlated. The results show that miR-101 overexpression inhibits the transformation of LC3-I to LC3-II and reduces cell proliferation, but increases p62/sqstm1 expression, apoptosis rate, and caspase-3 cleavage. However, co-expressing MALAT1 and miR-101 can eliminate the effect of miR-101 overexpression. This study confirmed that MALAT1 is overexpressed in CRC tissues and cell lines, and is positively correlated with LC3-II expression in CRC. However, introduction of miR-101 into CRC cells promoted cell proliferation and MALAT1-mediated autophagy and inhibited apoptosis^56^.

Autophagy activation of MALAT1 has been observed in tumors and in other diseases. For example, hypoxia can upregulate MALAT1 levels in endometrial cells in an HIF-1α-dependent manner during endometriosis, thereby activating autophagy. In addition, autophagy has a protective effect and can reduce apoptosis in endometriosis^57^.

## lncRNA H19

The lncRNA H19 gene is located on human chromosome 11 and is a maternally-expressed gene. LncRNA H19 activates autophagy by regulating the dual-specificity phosphatase 5 (DUSP5)-extracellular signal-regulated kinases1/2 (ERK1/2) axis in neuroblastoma. First, a rat model of middle cerebral artery occlusion (MCAO) was established. TTC(Triphenyltetrazolium chloride)-stained brain sections showed that H19 expression during brain ischemia/reperfusion (I/R) is about 35 times higher than expression in control samples. To investigate the role of H19 in neurons, the participants established an oxygen-glucose deprivation/reoxygenation (OGD/R) model of cells simulating I/R injury using SH-SY5Y neuroblastoma cells. The results showed that 8/24 h OGD induced significantly higher lncRNA H19 expression compared to the normal control group. In contrast, lncRNA H19 expression was not significantly changed during 12 and 24 h OGD reperfusion. The expression of lncRNA H19 was upregulated by cerebral I/R in rats and by OGD/R in vitro. Decreased lncRNA H19 expression or autophagy activation could protect cell damage induced by OGD/R. LncRNA H19 upregulation can activate autophagy after OGD/R injury, while transfecting H19 siRNA can inhibit autophagy. However, when rapamycin (RAP), an autophagy inducer, was added, autophagy inhibition by H19 siRNA was activated. These data show that lncRNA H19 activates autophagy during cerebral I/R injury^58^.

Similarly, Cui et al. found that H19 can induce hypoxia-reoxygenation injury by regulating autophagy in liver cancer cells^59^. Additional studies show that lncRNA H19 overexpression promotes invasion and autophagy *via* the phosphatidylinositol 3-kinase (PI3K)/protein kinase B (AKT)/mammalian target of rapamycin (mTOR) pathway in trophoblast cells^60^. Further, H19 decreases DNA methylation at the Beclin1 promoter region *via* the H19 S-adenosylhomocysteine hydrolase (SAHH)-DNA methyltransferase 3B (DNMT3B) axis, thereby enhancing autophagy^61^.

## lncRNA MEG3

In a study in 2019, Gao et al. explored the regulation of MEG3 on morphine-induced autophagy in HT22 mouse hippocampal neuronal cells. First, HT22 cells were treated with 10 µM morphine, which significantly increased c-fos, p/t-ERK, and p/t-protein kinase C (PKC) protein expression. These results suggest that morphine can enhance the expression of orexinergic receptor 1 (OX1R) and c-fos in HT22 cells and activate the ERK and PKC pathways. In addition, MEG3 expression significantly increased after treating HT22 cells with 10 µM morphine. To investigate the effect of MEG3 upregulation on morphine-induced autophagy in HT22 cells, the cells were transfected with si-MEG3, which sharply decreased MEG3 expression. Compared with the morphine + si-negative control (NC) group, Beclin-1 and LC3-II/LC3-I levels in the morphine + si-MEG3 group decreased. These results suggest that MEG3 upregulation is involved in morphine-induced autophagy in HT22 cells. Subsequently, HT22 cells were incubated with SCH772984, an ERK pathway inhibitor, which significantly reduced morphine-induced Beclin-1 and LC3-II/LC3-I expression, indicating that the ERK pathway plays an important role in HT22 cell autophagy. In addition, compared with the morphine + si-MEG3 group, Beclin-1 and LC3-II/LC3-I expression in HT22 cells in the morphine + si-MEG3 + SCH772984 group were further reduced. These experiments indicate that MEG3 upregulation may be involved in the effect of morphine on autophagy in HT22 cells by promoting ERK pathway activation^62^.

To study the role of MEG3 in glioma, Zhao et al. used a pcDNA-MEG3 lentiviral vector transfected into U251 cells. qRT-PCR analysis illustrated that compared with the negative control group, MEG3 expression was significantly higher. Flow cytometry analysis illustrated that the cell apoptosis rate was significantly increased after MEG3 overexpression. Meanwhile, immunofluorescence LC3 autophagy assays showed that LC3 was significantly increased in the MEG3 expression group. These data indicate that MEG3 expression can activate glioma cell autophagy^63^. Other researchers also found that MEG3 expression significantly inhibits glioma cell proliferation and promotes apoptosis and autophagy in vitro. Furthermore, MEG3 levels are negatively correlated with glioma malignancy, and can be used as an independent prognostic factor for glioma^63^.

## Other lncRNAs

In addition to the lncRNAs mentioned above, other lncRNAs play an important role in autophagy regulation. Many studies show that lncRNA plays a key role in regulating autophagy. lncRNA, as a ceRNA for miRNA, participates in the regulation of autophagy. For example, a previous study illustrated that downregulation of growth arrest-specific 5 (GAS5) regulates miR-23a expression, thereby influencing ATG3 and resulting in the inhibition of autophagy. Indeed, GAS5 overexpression inhibited mature miR-23a, but had no influence on pri-miR-23a (primary miR-23a) and pre-miR-23a (miR-23a precursor). Additionally, miR-23a can directly inhibit ATG3 expression in cells. Downregulating GAS5 inhibits the formation of LC3-II, ATG3, and the ATG5-ATG12 complex, thereby inhibiting autophagy. These data indicate that the GAS5/miR-23a/ATG3 axis may be a new regulatory network that could be helpful for understanding autophagy^64^.

A study of the lncRNA PVT1, in hepatic cellular cancer (HCC), showed that PVT1 lowers miR-365 and relieves miR-365-mediated ATG3 inhibition, resulting in autophagy^65^. In addition, long intergenic non-protein-coding RNA 665 (LINC00665) also plays a critical role in the development of liver cancer. Shan et al. found that LINC00665 regulates cell viability and autophagy through the miR-186-5p/mitogen-activated protein kinase kinase kinase kinase 3 (MAP4K3) axis in HCC^66^.

### The relationship between lncRNA and necroptosis

**Fig. 3:**
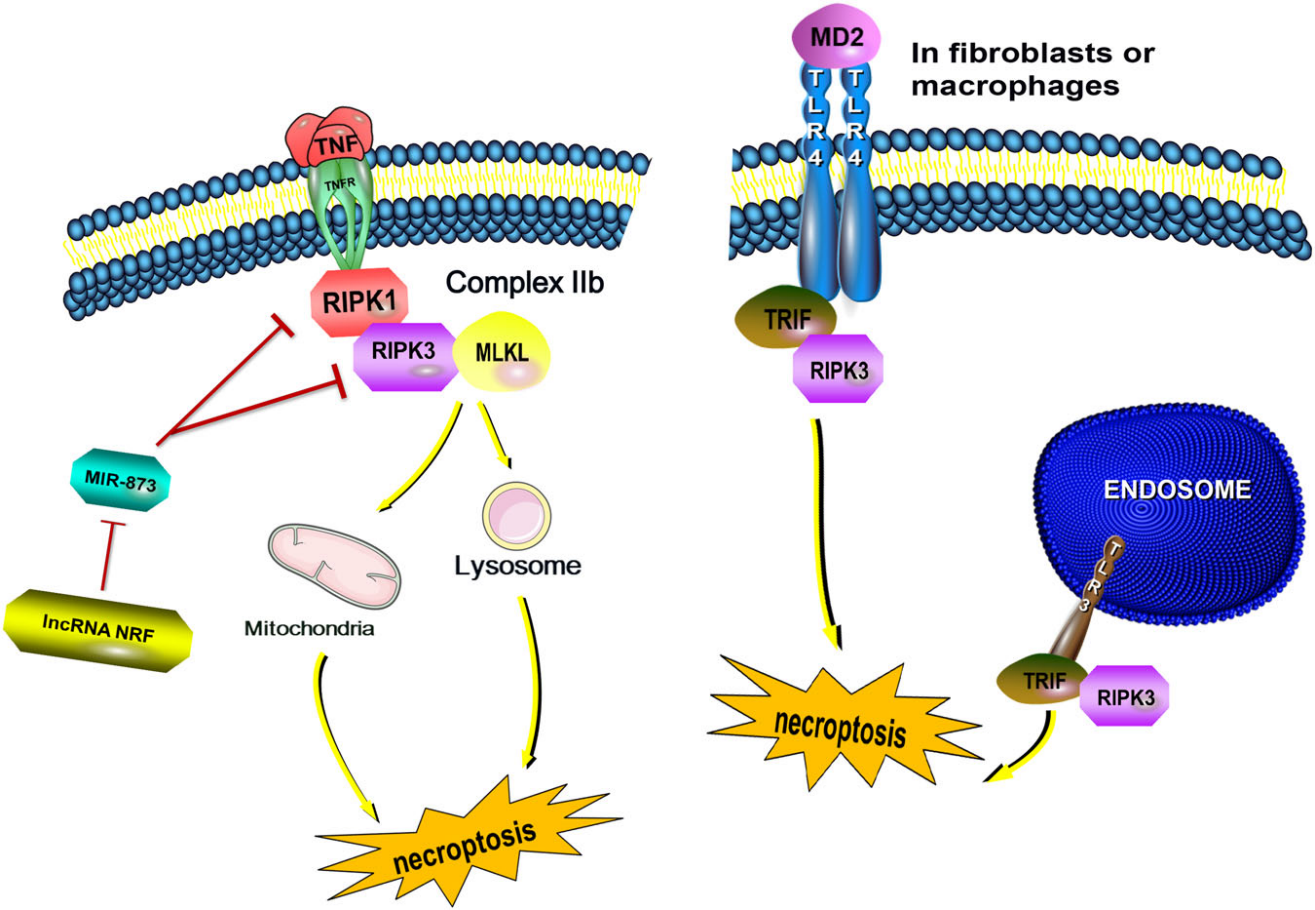
TNF-induced necroptosis. Necroptosis is a newly-described form of programmed cell death. Activating MLKL plays an important role in this process. The most widely studied subtype involves the TNF-inducible cell signal. In TNF-activated cells, RIPK1 is activated, and then activated RIPK1 forms complex IIb with RIPK3. MLKL is then phosphorylated by RIPK3 and recruited into the necrosome through interaction with RIPK3. By targeting organelles such as mitochondria and/or lysosomes, the cell membrane is destroyed, eventually leading to necrosis by cell lysis. LncRNA can regulate necroptosis by influencing this pathway. For example, the lncRNA NRF can directly downregulate miR-873, thereby reducing RIPK1/RIPK3 inhibition by miR-837, finally promoting necroptosis. In fibroblasts and macrophages, TLR3 or TLR4 interacts with RIPK3 through Total Recordable Incident Frequency (TRIF) to directly activate necrosis, independent from the RIPK1 signaling pathway. Cell swelling, mitochondrial dysfunction, membrane permeability, and cytoplasmic content release are observed.

Necroptosis is a newly identified form of PCD, and is mediated by several cell signaling pathways. These reactions activate mixed lineage kinase-like (MLKL) and lead to necroptosis^67^. Necroptosis has morphological characteristics such as cell swelling, mitochondrial dysfunction, increased membrane permeability, and cytoplasmic content release. Activating TNF and toll-like receptor (TLR) 3/TLR4 can lead to necroptosis. The most widely studied activation mechanism is the TNF-inducible cell signal, which relies on RIPK1, RIPK3, and MLKL^1,4,5^. In TNF-activated cells, RIPK1 is activated, which causes complex IIb binding with RIPK3. MLKL is then phosphorylated by RIPK3 and is recruited into the necrosome through interaction with RIPK3^5,68,69^. The necrosome targets organelles such as mitochondria and/or lysosomes, destroys the cell membrane, and eventually leads to necroptosis by cell lysis^5,70^. In fibroblasts and macrophages, TLR3 or TLR4 interacts with RIPK3 through TRIF, an integrin containing a TIR domain that induces interferon β interaction, to directly activate necroptosis. Importantly, this pathway is independent of the RIPK1 signaling pathway^71^ (Fig. 3).

Few studies describe the role of lncRNA in necroptosis. Two studies mentioned a type of lncRNA called TRINGS (Tp53-regulated inhibitor of necrosis under glucose starvation) in human tumor cells and suggested that p53 directly upregulates TRINGS in the absence of glucose^72,73^. TRINGS binds STRAP (serine–threonine kinase receptor-associated protein) and inhibits STRAP-GSK3β-NF-κB necrotic signaling to protect tumor cells from cell death. This is a novel necroptosis pathway that is different from the traditional necrotic pathway (RIPK1-RIPK3-MLKL). Another study showed that lncRNA Linc00176 affects the cell cycle and liver cancer cell survival by regulating the expression of more than 200 genes, such as miR-9 and miR-185. In HCCs that express Linc00176, these miRNAs are released from Linc00176 and downregulate their target mRNAs, which induce necroptosis of liver cancer cells^74^.

In addition, lncRNA interacts with miRNA and affects the expression of other miRNA species. Experiments have confirmed that the lncRNA necrosis-related factor (NRF) targets miR-873 and RIPK1/RIPK3 to regulate cardiomyocyte necroptosis^75^. After RIPK1 and RIPK3 were knocked down in cardiomyocytes, the number of H_2_O_2_-induced necrotic cells decreased significantly. This indicates that RIPK1 and RIPK3 are related to H_2_O_2_-induced cardiomyocyte necroptosis. After transfecting cardiomyocytes with miR-873 or negative control, RIPK1 and RIPK3 expression were analyzed by Western blotting, which confirmed that miR-873 inhibits RIPK1 and RIPK3 expression in cardiomyocytes. Furthermore, a miR-873 binding site in the RIPK1 or RIPK3 3’ UTR region (-UUCCUG-) is identified, and there is no binding site in mutant miR-873. Luciferase reporter gene assays showed that miR-873 overexpression resulted in a decrease in luciferase activity. The introduction of mutations in miR-873 greatly reduced the inhibitory effect of miR-873. Thus, miR-873 can directly bind to RIPK1 and RIPK3, thereby inhibiting H_2_O_2_-induced necroptosis. Bioinformatic analysis showed that lncRNA NRF contains a large number of miR-873 binding sites. RNA pull-down experiments showed that NRF was pulled down by biotinylated wild-type miR-873, but mutations that disrupt the base pairing between NRF and miR-873 inhibited NRF pull-down, indicating that the recognition sequence is specific. In addition, we used a biotin-labeled NRF probe to detect the location of the interaction between NRF and miR-873. The results showed that miR-873 can be co-precipitated by NRF in the cytoplasm. These results show that NRF can directly bind to miR-873 and downregulate the level of miR-873. Further, miR-873 inhibits the translation of RIPK1/RIPK3 in cardiomyocytes and inhibits RIPK1/RIPK3-mediated necroptosis. These results show that lncRNA can be combined with related proteins in the PCD pathway or as ceRNA combined with related miRNAs to regulate necroptosis. Compared with autophagy and apoptosis, the interaction between lncRNAs and necroptosis remains to be further explored.

### The relationship between lncRNA and ferroptosis

It is now recognized that ferroptosis is a non-apoptotic form of cell death. The main reason for death is the accumulation of iron-dependent ROS and the consumption of plasma membrane polyunsaturated fatty acids. The morphological characteristics of cellular ferroptosis show that the nucleus is normal, but the mitochondria are smaller than normal, the membrane density is increased, the outer membrane is ruptured, and mitochondria is shrunken or absent^76,77^.

**Fig. 4:**
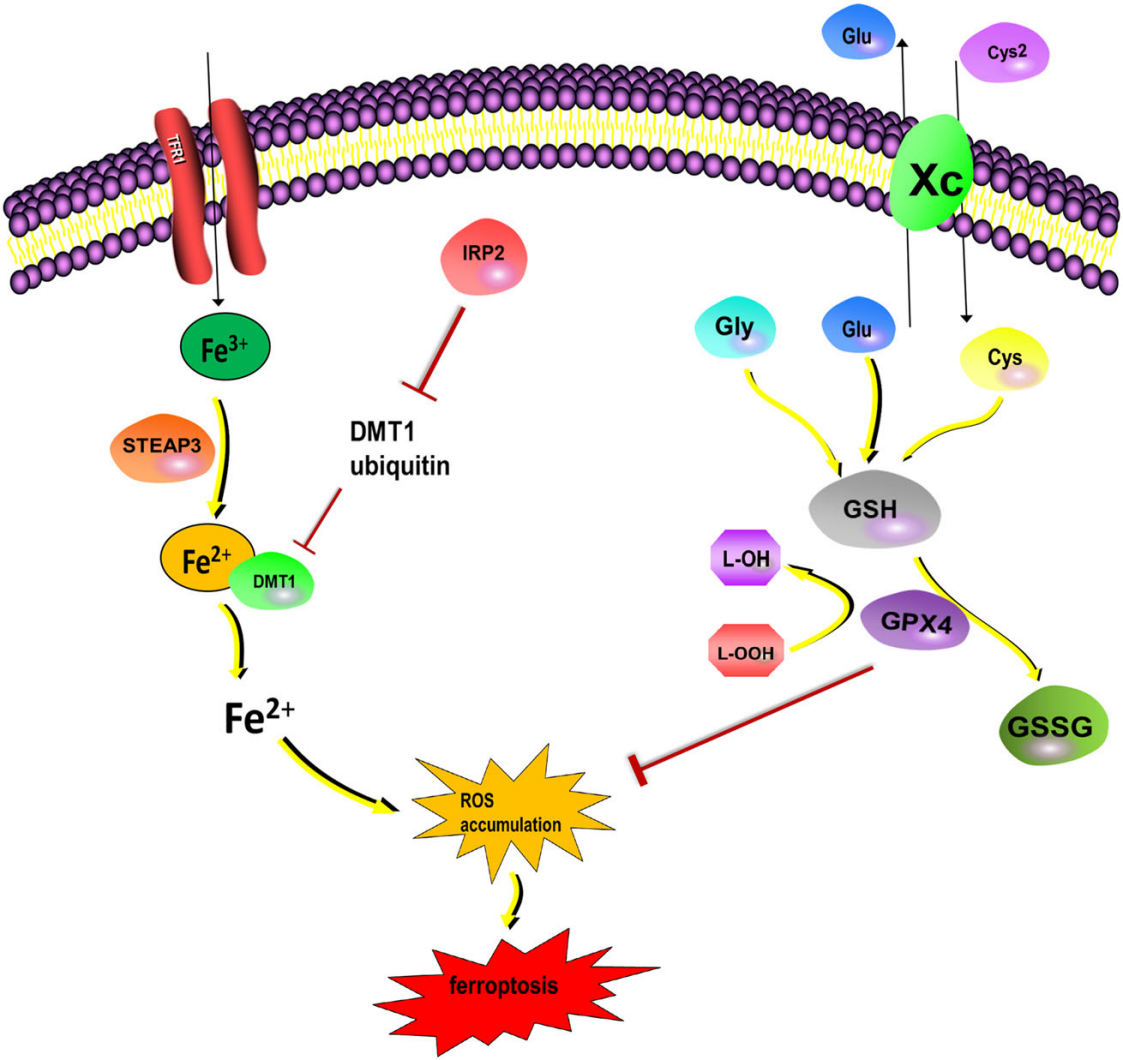
Cellular ferroptosis. The related lipid peroxidation pathway mediated by GPX4 plays an important role in inhibiting ferroptosis. System Xc—is a cystine/glutamate antiporter that transports intracellular Glu to the outside of the cell and transfers extracellular Cys2 into the cell. Then, Cys2 is converted into Cys for the synthesis of GSH. GPX4 converts GSH into oxidized GSSG and reduces cytotoxic lipid peroxide (L-OOH) to the corresponding alcohol (L-OH), which ultimately reduces ROS accumulation. On the other hand, Fe^3+^ enters the cell and is reduced to Fe^2+^ by the iron oxidoreductase STEAP3. Then, DMT1 causes Fe^2+^ release from the endosome, leading to ROS accumulation, which in turn leads to lipid peroxidation and ferroptosis. IRP2 (IREB2) can inhibit the ubiquitination of TfR1 and divalent metal transporter 1 (DMT1) to increase cellular iron uptake and promote ferroptosis.

Cellular ferroptosis occurs roughly as follows: System Xc—is a cystine/glutamate antiporter, which transports intracellular glutamate (Glu) to the outside of the cell and transfers extracellular cystine (Cys2) into the cell. Cys2 is converted into cysteine (Cys) for the synthesis of glutathione (GSH)^78^. GSH peroxidase 4 (GPX4) converts GSH into oxidized glutathione (GSSG) and reduces cytotoxic lipid peroxide (L-OOH) to the corresponding alcohol (L-OH), which ultimately reduces the accumulation of ROS^79^. In addition, some studies show that p53 is an important tumor suppressor, exerting tumor-suppressive effects by inhibiting ferroptosis^80^ (Fig. 4).

Circulating iron (Fe^3+^) enters the cell and is reduced to Fe^2+^ by the iron oxidoreductase STEAP3 (six-transmembrane epithelial antigen of the prostate 3). Next, Fe^2+^ is released from the endosome by divalent metal transporter 1 (DMT1) and is transported into the unstable iron pool in the cytoplasm, leading to ROS accumulation, which leads to lipid peroxidation and ferroptosis. Iron regulatory protein 2 (IRP2, also known as IREB2) can inhibit the ubiquitination of transferrin receptor 1 (TfR1) and DMT1 to upregulate cellular iron uptake and promote ferroptosis^81^. Recent studies showed that ferritin inhibitor 1 (FSP1) (previously known as flavoprotein apoptosis-inducing factor mitochondrial-related 2, AIFM2) is a non-GPX4-dependent ferroptosis inhibitor^82–84^.

Wang et al. found that the lncRNA LINC00336 inhibits ferroptosis in lung cancer by acting as a ceRNA^85^. First, the researchers used RSL3 (a ferroptosis activator) to treat A549 and SPC-A-1 lung cancer cells and observed that LINC00336 overexpression limits RSL3-induced cellular ferroptosis. Further, LINC00336 overexpression in A549 or SPC-A cells can resist ferroptosis caused by protein kinases in a dose- and time-dependent manner. In addition, LINC00336 overexpression significantly reduced intracellular Fe^2+^, lipid ROS, and intracellular mitochondrial superoxide concentrations. These results indicate that LINC00336 overexpression downregulates the occurrence of ferroptosis.

Regarding ferroptosis, several studies show that lncRNA LINC00336 inhibits ferroptosis of lung cancer cells by acting as a ceRNA. Additional experiments confirmed that LINC00336 overexpression significantly enhanced the expression of cystathionine β synthase (CBS), a ferroptosis marker, in A549 and SPC-A-1 cells, while knocking down LINC00336 significantly slowed down the growth of PC9 cell (a lung cancer cell line)^85^. The expression of CBS in cells indicates that LINC00336 can positively regulate CBS levels, thereby affecting ferroptosis. In addition, transfected MIR6852 can directly bind to LINC00336, and among the binding sites, the 3′-MIR6852 binding site on LINC00336 has a higher binding activity than the 5′-MIR6852 binding site. Finally, a lentivirus was used to introduce MIR6852 and spongy MIR6852 into SPC-A-1 cells. MIR6852 overexpression increased RSL3-induced ferroptosis, while spongy MIR6852 reduced RSL3-induce ferroptosis. These results show that MIR6852 increases cellular ferroptosis and increases the intracellular concentration of lipid ROS, iron, Fe^2+^, and mitochondrial superoxide. Additionally, MIR6852 directly binds to LINC0033 and inhibits CBS-mediated inhibition of ferroptosis, ultimately increasing overall cellular ferroptosis.

Regulation of the p53 tumor suppressor pathway by lncRNA is a topic of great interest. Mao et al. showed that the p53-related lncRNA P53RRA can directly interact with the functional domain of the signal protein in the cytoplasm by activating the p53 pathway to promote ferroptosis and act as a cancer suppressor^86^. In more detail, P53RRA combines with Ras GTPase-activating protein-binding protein 1 (G3BP1), using nucleotides 1 and 871 of P53RRA and the RRM interaction domain of G3BP1 (aa177-466). Another study showed that p53 plays a negative role in the regulation of iron oxidation. Spermidine/spermidine N1-acetyltransferase 1 (SAT1) is a transcriptional target of p53^87^. The expression of SAT1 induces lipid peroxidation and ferroptosis during ROS-induced stress.

In addition, the lncRNA P53RRA can act on the G3BP1 protein complex to promote p53 removal and regulate ferroptosis.

## Conclusion

Recent studies on lncRNAs have shown that lncRNAs play an important role in regulating gene expression, and they also participate in the regulation of PCD, including autophagy, apoptosis, necroptosis, and ferroptosis. LncRNA can inhibit or promote PCD by directly or indirectly regulating protein complexes and microRNAs. Indeed, ceRNAs of mRNA are important regulatory pathways. In terms of clinical significance, lncRNAs can regulate PCD in cancer cells^88–90^.

Several studies have confirmed that lncRNAs affect cancer cell growth in cancer patients, which important for clinical treatment and cancer prognosis. LncRNA plays an important role in drug resistance in breast, gastric, and lung cancer. For instance, lncRNA MALAT1 can inhibit breast cancer metastasis^90^. MALAT1 overexpression is positively correlated with tumor progression and metastasis of various tumor types, including breast cancer. In addition, lncRNA LINC00673 regulates the proliferation, migration, invasion, and epithelial-mesenchymal transition of non-small cell lung cancer by inhibiting miR-150-5p^91^. In digestive system cancer, the lncRNA MIF-AS1 promotes cancer cell proliferation and reduces apoptosis^92^. The lncRNA UICLM (upregulated in colorectal cancer liver metastasis) regulates zinc finger E-box binding homeobox 2 (ZEB2) expression by acting as a ceRNA of microRNA-215, thereby promoting liver metastasis of colorectal cancer^93^. Cancer is an important public health concern. An in-depth understanding of lncRNAs will provide new targets for cancer treatment and improve clinical treatment of the disease.
